# Older adults, clinicians, and researchers’ preferences for measuring adherence to resistance and balance exercises

**DOI:** 10.1186/s12877-023-04237-x

**Published:** 2023-08-30

**Authors:** Caitlin McArthur, Gabriella Duhaime, David Gonzalez, Nanna Notthoff, Olga Theou, Scott Kehler, Adria Quigley

**Affiliations:** 1 Dalhousie University, 5869 University Avenue, B3H 1X7 Halifax Nova Scotia, Canada; 2 Faculty of Sport Science, Leipzig University, Jahnalleee 59, 04109 Leipzig, Germany; 3 5869 University Avenue, B3H 1X7 Halifax Nova Scotia, Canada

**Keywords:** Exercise, Adherence, Balance, Resistance training, Resistance, Strength training

## Abstract

**Background:**

Resistance and balance training are important exercise interventions for older populations living with chronic diseases. Accurately measuring if an individual is adhering to exercises as prescribed is important to determine if lack of improvement in health outcomes is because of issues with adherence. Measuring adherence to resistance and balance exercises is limited by current methods that depend heavily on self-report and are often better at and tailored towards capturing aerobic training parameters (e.g., step count, minutes of moderate to vigorous physical activity). Adherence measures must meet users’ needs to be useful.

**Methods:**

Using a Dillman tailored study design, we surveyed researchers who conduct exercise trials, clinicians who prescribe exercise for older adults, and older adults to determine: (1) how they are currently measuring adherence; (2) barriers and facilitators they have experienced to measurement; and (3) the information they would like collected about adherence (e.g., repetitions, sets, intensity, duration, frequency, quality). Surveys were disseminated internationally through professional networks, professional organizations, and social media. Participants completed an online survey between August 2021 and April 2022.

**Results:**

Eighty-eight older adults, 149 clinicians, and 41 researchers responded to the surveys. Most clinicians and researchers were between the ages of 30 and 39 years, and 70.0% were female. Most older adults were aged 70–79 years, and 46.6% were female. Diaries and calendars (either analog or digital) were the most common current methods of collecting adherence data. Users would like information about the intensity and quality of exercises completed that are presented in clear, easy to use formats that are meaningful for older adults where all data can be tracked in one place. Most older adults did not measure adherence because they did not want to, while clinicians most frequently reported not having measurement tools for adherence. Time, resources, motivation, and health were also identified as barriers to recording adherence.

**Conclusions:**

Our work provides information about current methods of measuring exercise adherence and suggestions to inform the design of future adherence measures. Future measures should comprehensively track adherence data in one place, including the intensity and quality of exercises.

**Supplementary Information:**

The online version contains supplementary material available at 10.1186/s12877-023-04237-x.

## Background

Exercise has numerous health benefits and is a common component of guidelines for the prevention and management of conditions that affect older adults. For example, resistance and balance training are an important part of exercise interventions for several aging-related chronic conditions including arthritis, [1] osteoporosis, [2] sarcopenia, [3] and frailty [4]. Indeed, resistance training is essential to prevent frailty and sarcopenia, while administering a challenging balance training regime alone can decrease the risk for falls for community dwelling older adults by up to 42% [5].

The effectiveness and efficacy of any exercise intervention to induce change in health outcomes depends heavily on adherence to the intervention. In the context of exercise, adherence is a person’s correct level of completion of the recommended training regimen and treatment (e.g., number of days per week exercising) [6] including the degree to which an individual conforms to the prescribed exercise dosage (e.g., as determined by heart rate, rate of perceived exertion, percentage of 1-repetition maximum) [7]. Accurate measurement of adherence is essential in exercise studies and in clinical work to elucidate whether improvements (or lack thereof) can be attributed to poor adherence with the prescribed intervention and dose [8].

Measuring exercise adherence often relies on self-report, which can suffer from recall and social desirability biases, [9] and often places emphasis on aerobic variables (e.g., step count, minutes of moderate to vigorous aerobic activity). A recent systematic review of adherence to therapeutic exercise for musculoskeletal pain found that the most commonly used parameter to assess adherence was self-reported frequency of exercise (e.g., number of days per week) via exercise logs [10]. In the same review, adherence to exercise dose was only measured in 15% of the included studies, with 9% measuring it via self-report and 5% measuring it objectively [10]. The majority of objectively measured variables pertained to aerobic outcomes (i.e., minutes and intensity of walking or running), [10] and relied on accelerometers. Accelerometers are not able to capture all metrics of complex, dynamic,three-dimensional movements often included in other types of exercise like resistance and balance training (e.g., squats, lunges, dancing, tai chi, yoga). [11] Likewise, they do not easily capture the intensity of resistance and balance exercises (e.g., working at a certain percentage of 1-repetition maximum, balance exercises that perturb the person’s centre of mass). [11, 12].

Previous work has also identified that measures of exercise adherence have poor conceptual underpinnings and are not fit for purpose, often relying on pure quantification of exercise completion without considering the quality of completion [8, 13]. For example, a recent consensus process in the United Kingdom deemed six common exercise adherence measures were not appropriate for clinical research or practice based on suitability, relevance, acceptability, or appropriateness [14]. Further, important outcomes varied between patients, carers, and health professionals [15] Simple quantification of exercise completion was important but insufficient according to patients and physiotherapists, while researchers were most interested in simple quantification [14]. This discrepancy highlights the usefulness of multiple stakeholder involvement in the future development of adherence measures suitable for research and clinical practice.

Exercise adherence must be clearly conceptualized to facilitate appropriate and robust assessment [14]. Given the inability of currently available tools to measure exercise adherence appropriately and the need to include multiple stakeholders’ perspectives, we explored the perspectives of researchers, clinicians, and older adults regarding exercise adherence measurement. Specifically, we surveyed researchers who conduct exercise trials, clinicians who prescribe exercise for older adults, and older adults to determine: (1) how they are currently measuring adherence; (2) barriers and facilitators they have experienced to measurement; and (3) the information they would like collected about adherence (e.g., repetitions, sets, intensity, duration, frequency, quality). We focused on resistance and balance training because of their importance for multiple health conditions, the relative complexity of measuring adherence for these types of exercise, and because tools to measure adherence to aerobic exercise already exist. The information gained from this study can be used to inform development of new exercise adherence measures that meet the needs of users and overcome barriers to their use.

## Methods

***Participants***: We had three target populations: (1) researchers who have completed exercise studies incorporating resistance and balance training as part of their intervention for adults 50 years or older; (2) clinicians who incorporate resistance and balance exercises in their practice for adults aged 50 years or older; and (3) adults aged 50 years or older who complete resistance and/or balance exercises. We chose 50 years old as our cut-off score as we wanted to be inclusive of individuals entering older age who may still be active [16].

We targeted our recruitment based on our three populations of interest and deployed surveys locally, nationally, and internationally. As we had collaborators in Germany and Canada, our survey was distributed in English and German. Based on a comprehensive literature review and through professional networks of the co-investigators, we compiled a list of researchers who have completed exercise intervention studies including balance and resistance training. An email invitation to participate in the study was sent to these researchers. To recruit clinicians, we disseminated the recruitment poster and survey link through organizations such as the Canadian Physiotherapy Association, International Association of Physiotherapists Working with Older People, Canadian Society of Exercise Physiologists, World Physiotherapy, and Canadian Kinesiology Alliance. Likewise, to recruit older adults, we disseminated the study information through organizations such as the Canadian Association of Retired Persons. We also posted the recruitment poster and link to the survey on social media (e.g., Twitter, LinkedIn), tagging and mentioning relevant organizations as described above. To encourage participation and as a token of gratitude for completion, participants who completed the survey were entered for a chance to win a $25 gift card for an online retailer. Three follow-up reminders were sent to all groups and social media ads were re-posted every other week to complete the survey. Surveys were collected between August 2021 and April 2022.

### Survey development and structure

We developed our survey questions following a Dillman tailored study design [17]. We developed three separate surveys, one each for researchers, clinicians, and older adults to ensure we asked questions specific to their needs and uses of exercise adherence measures. Each survey consisted of a mixture of closed-ended, ordinal scale, and open-ended questions. The researcher and clinician surveys consisted of three sections exploring how current exercise adherence was measured, barriers and facilitators to current measurement, and what information they would like measured. A priori, we hypothesized that researchers and clinicians may want to measure a good quality set or repetition of an exercise to capture adherence. Thus, we also asked a question about how they would define a good set or repetition. The older adult survey began with the Physical Activity Scale for the Elderly (PASE) to gain an understanding of the activity level of participants. PASE is a brief, self-administered questionnaire where participants recall their physical activity over the past 7 days [18]. The PASE assigns a score based on frequency, duration, and intensity level of walking, recreational activities, exercise, housework, yard work, and caring for others [18]. Values range from 0 to 793, where higher scores indicate higher levels of physical activity [18]. After completing the PASE, the survey branched based on whether participants participated in resistance or balance training, or both. If participants completed resistance and balance training, the survey questions explored if and how they currently recorded these exercises, what information they would like to record, and how they would like to record it. If participants did not complete resistance and balance training, the questions explored why they did not complete this training (barriers) and how recording their exercise adherence might help them complete it (facilitators). Prior to survey deployment, we pilot tested the survey with 5 people for readability, ease of navigation, and estimated completion time. The surveys were administered online through Opinio software. The full surveys can be found in Supplementary File 1.

### ***Data Analysis***

Results of closed-ended questions are presented as frequencies and percentages of the participants who answered each question. Open-ended questions were analyzed using inductive thematic content analysis [19] by one researcher to identify common themes in the responses. For streamlined results presentation, we combined the results for survey questions asking about the most and least important items to collect for adherence monitoring in balance and strength training by presenting frequency of responses for these two questions together. The denominators remained as the number of participants who answered each individual question. All survey responses that were started were included in analysis. Data were analyzed in Opinio and Excel.

### Ethical approval and consent to participate

This study was reviewed and approved by the Research Ethics Board at Dalhousie University. All methods were carried out in accordance with the relevant guidelines and regulations. Informed consent was obtained from all participants and/or their legal guardian.

## Results

Table 1 provides a summary of the demographic characteristics of participants and completion rates of the surveys. There were 88, 149, and 41 responses to the surveys for older adults, clinicians, and researchers, respectively. Most clinicians and researchers were between the ages of 30 and 39 years, and 70.0% were female. Most older adults were aged 70–79 years, and 46.6% were female. The average PASE score was 206, indicating a cohort of older adults with low to moderate activity levels. Overall, 50.4% of surveys that were started were completed, with the average survey being 55.1% complete.

**Table 1:** Sample demographics and completion rate

	Older adults n = 88	Clinicians n = 149	Researchers n = 41
Age, n (%)			
20–29 years 30–39 years 40–49 years 50–59 years 60–69 years 70–79 years 80–89 years Prefer not to respond	- - - 9 (10.2) 14 (15.9) 26 (29.5) 5 (5.7) 2 (2.27)	24 (16.1) 35 (23.5) 23 (15.4) 30 (20.1) 18 (12.1) 1 (0.7) 2 (1.3) -	7 (17.1) 14 (34.1) 7 (17.1) 7 (17.1) 2 (4.9) - - -
Female, n (%)	41 (46.6)	110 (73.8)	23 (56.1)
% who completed survey, n (%)	50 (56.8)	75 (50.3)	15 (36.6)
% of survey complete, mean (standard deviation)	62.6 (45.3)	58.8 (43.3)	44.0 (42.9)

### Current measurement of adherence

Table 2 summarizes responses to the questions about current measurement of adherence to resistance and balance exercises. Clinicians and researchers most frequently used a calendar or diary (pen and paper or digital) to measure adherence for both resistance and balance exercises. Older adults most frequently did not measure adherence for either resistance or balance exercises, but when they did, they most frequently used a pen and paper or digital calendar or diary. Nearly one quarter of responding clinicians reported using another way to measure adherence, which most frequently was attendance at a group class or through self-report from their client or patient. For resistance exercises, older adults most frequently collected information about total exercise time and the amount of weight used, while clinicians and researchers most frequently collected number of repetitions and sets, and the number of days completed. Very few older adults reported recording balance exercises, while clinicians and researchers most frequently collected the types, difficulty, and number of days balance exercises were completed. Older adults most frequently reported not measuring adherence to both resistance and balance exercises because they did not want to, while clinicians most frequently reported not having measurement tools for adherence.

**Table 2:** Current measurement of adherence to strength and balance exercises

	Older adults	Clinicians	Researchers
Survey question	n (%)	n (%)	n (%)
How do you currently measure adherence for strength exercises? (Check all that apply)			
Calendar/diary (pen/paper or app)	12 (13.6)	77 (51.7)	28 (68.3)
Phone call from clinic or study team	n/a	6 (4.0)	9 (22.0)
Wearable device (e.g., Fitbit)	11 (12.5)	n/a	n/a
Do not measure	22 (25.0)	31 (20.8)	6 (14.6)
Other	5 (5.7)	41 (27.5)	1 (2.4)
What data do you currently collect to report adherence to strength exercises? (For those who do report adherence, check all that apply)			
Number of repetitions	11 (12.5)	46 (30.9)	10 (24.4)
Number of sets	9 (10.2)	41 (27.5)	10 (24.2)
Number of repetitions with good form and at a selected intensity	3 (3.4)	24 (16.1)	4 (39.0)
Number of sets and/or repetitions with good form and at a selected intensity	1 (1.1)	18 (12.1)	4 (39.0)
Total exercise time	18 (20.5)	21 (14.1)	7 (17.1)
Time to complete a set	1 (1.1)	5 (3.4)	0 (0)
Time to complete a repetition	1 (1.1)	4 (2.7)	1 (2.4)
Intensity of repetitions (e.g., % of 1-rep max)	1 (1.1)	5 (3.4)	2 (4.9)
Rate of perceived exertion	n/a	31 (20.8)	8 (19.5)
Number of different exercises	4 (4.5)	33 (22.1)	10 (24.4)
Number of days completed	9 (10.2)	49 (32.9)	14 (66.7)
Amount of weight used	15 (17.0)	n/a	n/a
Raw data – e.g., individual joint velocities, angles, accelerations	n/a	3 (2.0)	0 (0)
Other	5 (5.7)	9 (6.0)	2 (4.9)
Why don’t you measure adherence to strength exercises? (For those who do not record)			
Not enough time	n/a	2 (1.3)	2 (4.9)
I don’t want to	15 (17.0)	n/a	1 (2.4)
I don’t know how to	2 (2.3)	2 (1.3)	0 (0)
I don’t have the tools to record them	1 (1.1)	9 (6.0)	0 (0)
Other	3 (3.4)	7 (4.7)	0 (0)
How do you currently measure adherence for balance exercises? (Check all that apply)			
Calendar/diary (pen/paper or app)	3 (3.4)	53 (35.6)	14 (66.7)
Phone call from clinic or study team	n/a	5 (3.4)	4 (39.0)
Wearable device (e.g., Fitbit)	1 (1.1)	n/a	n/a
Do not measure	25 (28.4)	17 (11.4)	5 (12.2)
Other	2 (2.3)	27 (18.1)	3 (7.3)
What data do you currently collect to report adherence to balance exercises? (For those who do report adherence, check all that apply)			
Difficulty of balance exercises	n/a	44 (29.5)	7 (17.1)
Total exercise duration	1 (1.1)	22 (14.8)	3 (7.3)
Number of different exercises	1 (1.1)	36 (24.2)	1 (2.4)
Type of balance exercises	2 (2.3)	45 (30.2)	5 (12.2)
Number of repetitions	2 (2.3)	37 (24.8)	5 (12.2)
Number of sets	1 (1.1)	25 (16.8)	5 (12.2)
Number of days completed	1 (1.1)	42 (28.2)	10 (24.4)
Other	2 (2.3)	6 (4.0)	1 (2.4)
Why don’t you measure adherence to balance exercises? (For those who do not report adherence)			
Not enough time	n/a	1 (0.7)	1 (2.4)
I don’t want to	11 (12.5)	1 (0.7)	1 (2.4)
I don’t know how to	3 (3.4)	2 (1.3)	0 (0)
I don’t have the tools to record them	5 (5.7)	7 (4.7)	0 (0)
Other	5 (5.7)	4 (2.7)	3 (7.3)

*Values do not add up to 100% because of response rate for individual questions

**Fig. 1:**
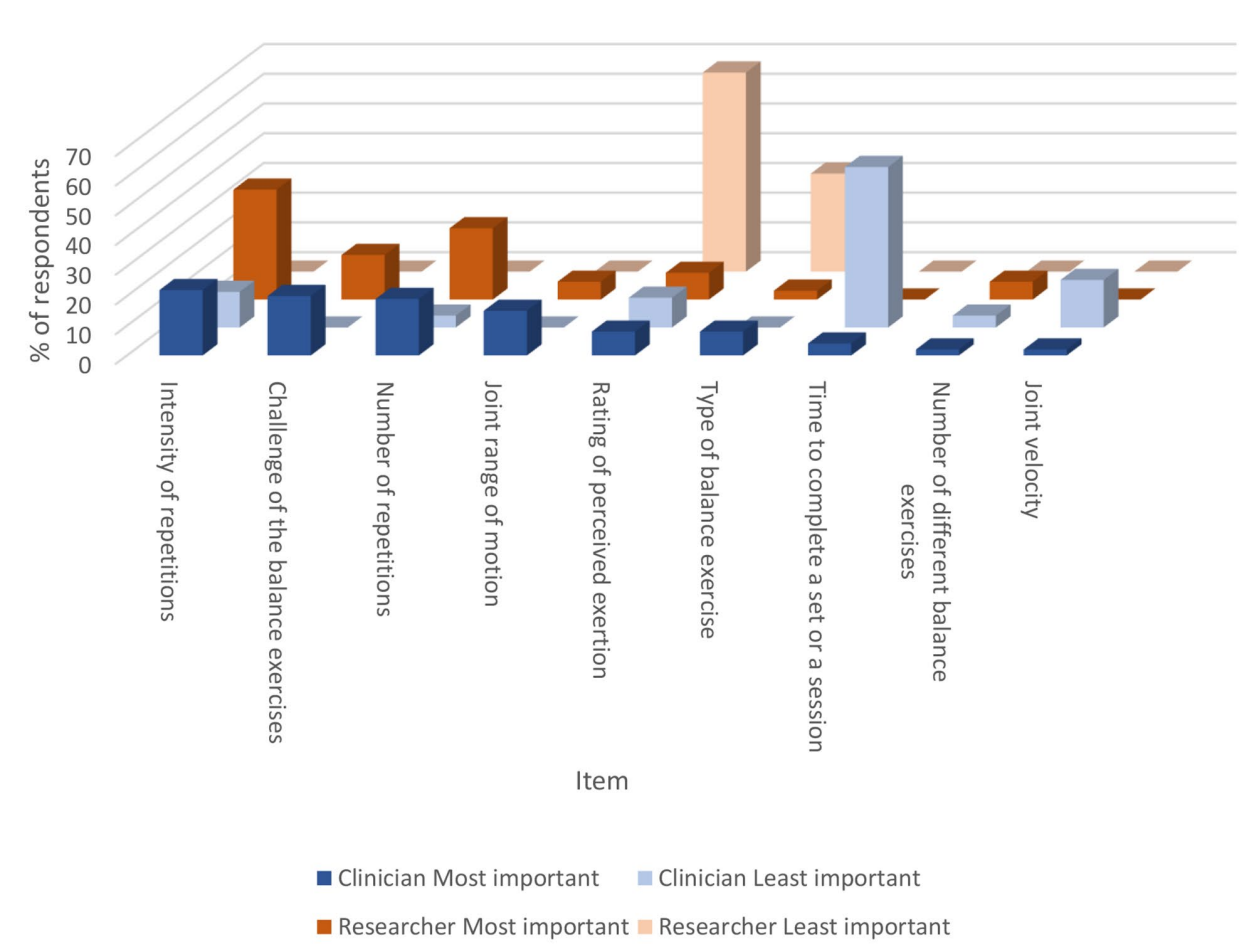
Ranking for what constructs define a good repetition or set of balance and strength exercises

Figure 1 displays the most and least important items ranked by clinicians and researchers to characterize parameters of a good quality set or repetition of a balance or resistance exercise. The top three most important items were intensity of repetitions, challenge of the exercise, and number of repetitions for both clinicians and researchers, while time to complete a repetition and rating of perceived exertion were most frequently rated as least important by clinicians and researchers, respectively.

### Barriers and facilitators to measuring adherence

Based on responses from open-ended questions, older adults identified an easy and functional tool, either pen and paper or a device/app, as a facilitator for measuring adherence. Having someone show them how to record their exercise was also highlighted as something that would make it easier for them to measure adherence. Barriers include a lack of time, resources, and motivation, not being able to record everything in one location and not being familiar with the exercises.

Clinicians and researchers identified clear, specific instructions and salience to clients as facilitators to exercise adherence measures. Like older adults, a lack of time and resources were repeatedly identified as barriers, while patient motivation level and health also impacted adherence measures. Other barriers include unreliable patient self-report and a lack of a simple, accurate measurement tool with real-time feedback for clients.

### Information participants would like collected to measure adherence

**Fig. 2:**
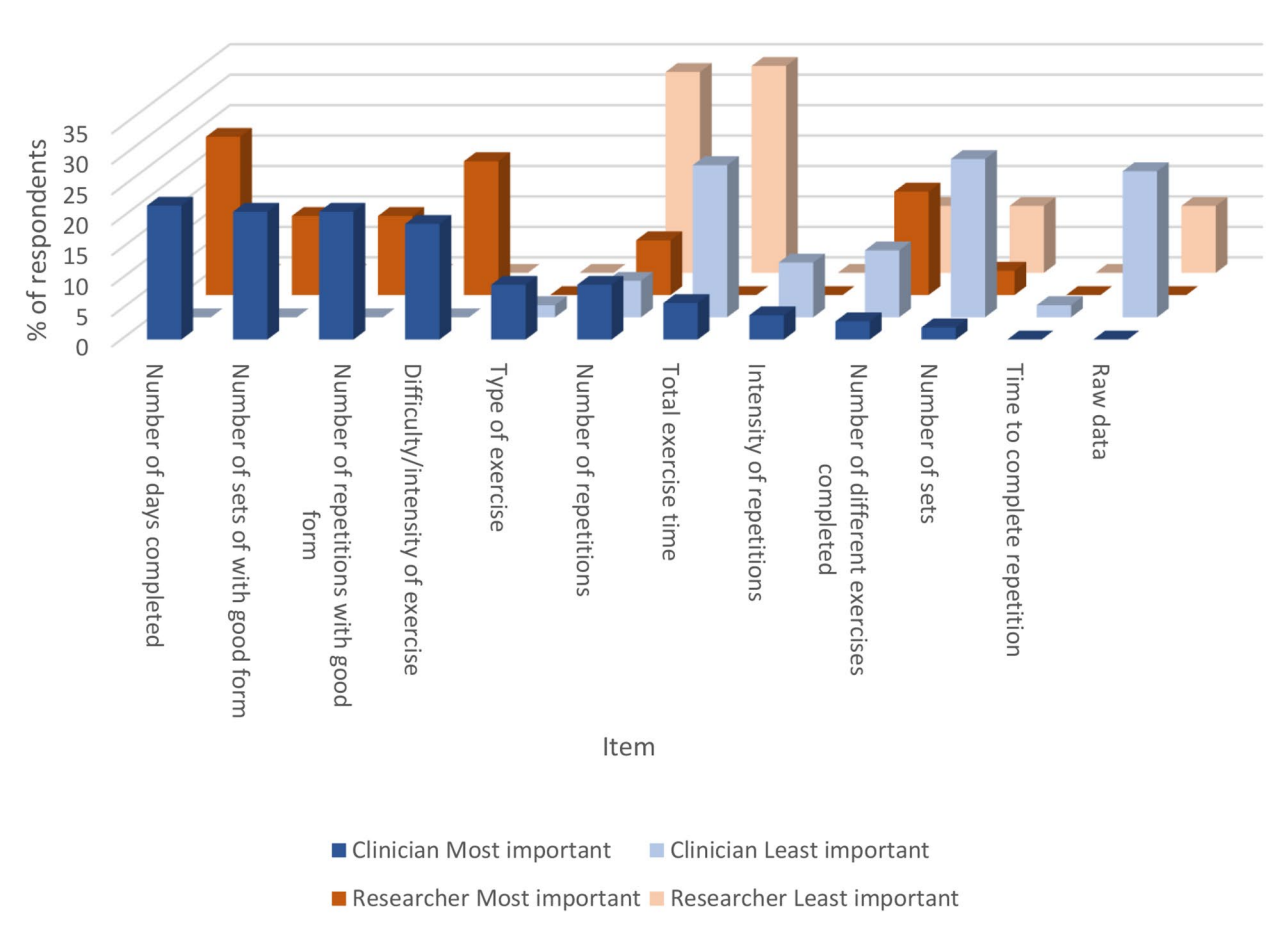
Ranking for most and least important items that clinicians and researchers would like to measure for strength and balance adherence

Figure 2 shows the most and least important items that clinicians and researchers would like to measure for resistance and balance exercise adherence.

The constructs that were most frequently rated as most important were number of repetitions with good form, number of days, and difficulty or intensity of the exercise for both clinicians and researchers. For least important, number of sets was most frequent for clinicians and total exercise time was second most frequent and most frequent for clinicians and researchers, respectively. Other information that clinicians and researchers would like information about includes quality and form of exercises, rating of perceived exertion, how the exercises translate to functional abilities, client pain levels, client perceptions of exercise intensity, benefit, and enjoyment. For balance exercises specifically, clinicians requested data regarding number of falls and adverse events, along with client progression with exercises.

**Fig. 3:**
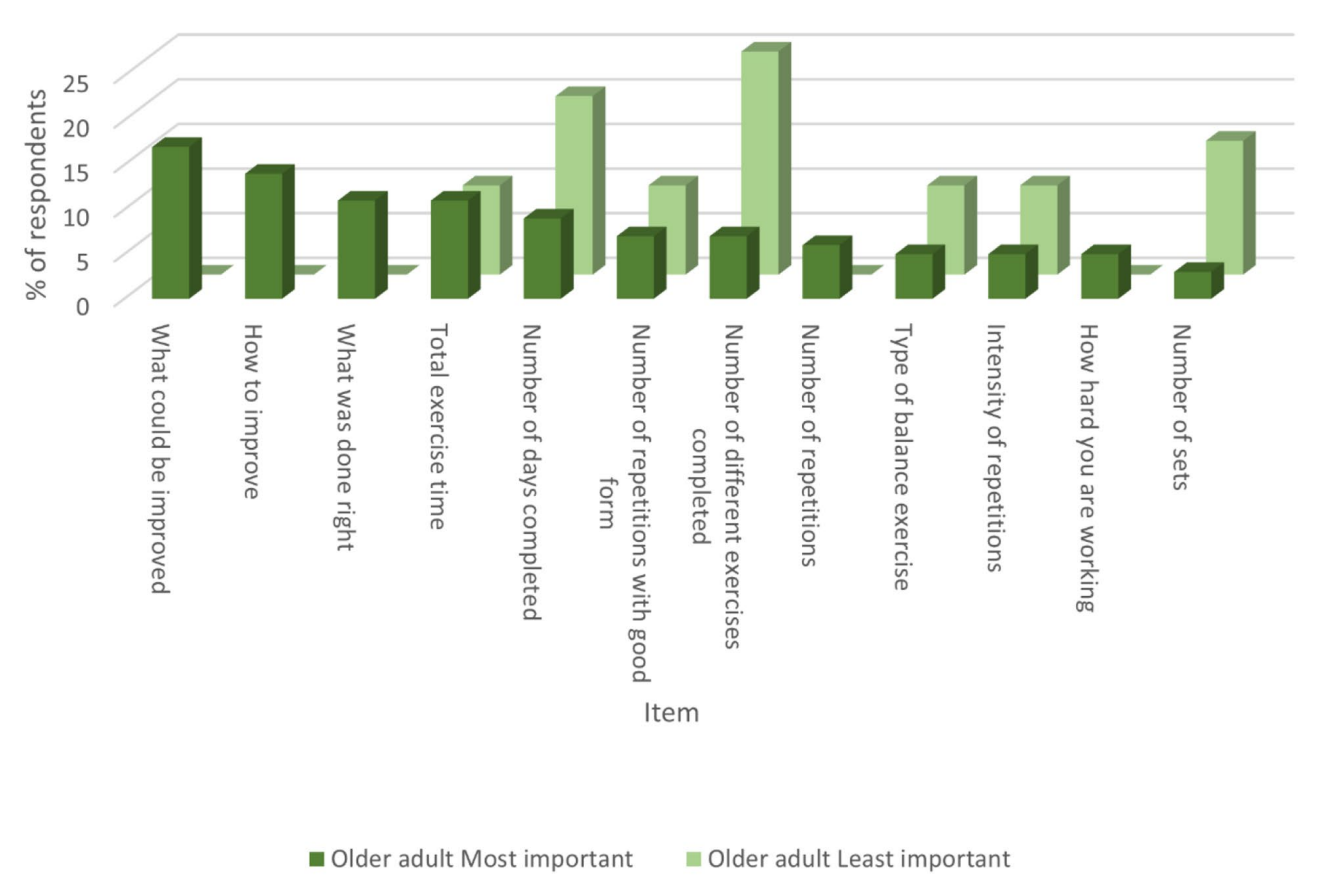
Ranking for what items older adults would like reported to them when measuring adherence of balance and strength exercises

Figure 3 displays the results of what items older adults ranked as most and least important to be reported to them regarding adherence to resistance and balance exercises. The items most frequently ranked as most important were what the older adult could improve upon, how they can improve, and what they did correctly. Other information that older adults would like to be collected about their exercises includes heart rate, VO2max, and walking speed and distance.

Older adults (n = 23, 26.1%), clinicians (n = 32, 21.5%), and researchers (n = 7, 17.1%) most frequently rated a cell phone as the mobile device they would be most comfortable using. Many older adults (n = 10, 11.4%) selected “other” and wrote “pen and paper” as their preference. For specifications of the software, Clinicians and researchers most frequently wanted to know the reliability (clinicians: n = 52, 34.9%; researchers: n = 15, 36.6%), validity (clinicians: n = 49, 32.9%; researchers: n = 13, 31.7%), and responsiveness (clinicians: n = 45, 30.2%; researchers: n = 10, 24.4%) for measuring adherence.

## Discussion

We surveyed older adults, clinicians, and researchers to identify current measures of adherence for resistance and balance exercises, barriers and facilitators to their use, and what constructs users would like in future measures. We found that diaries and calendars (either analog or digital) were the most common current method of collecting adherence data. Users would like information about the intensity and quality of exercises completed that are presented in clear, easy to use formats that are meaningful for older adults where all data can be tracked in one place. Most older adults did not measure adherence because they did not want to, while clinicians most frequently reported not having measurement tools for adherence. Time, resources, motivation, and health were also identified as barriers to recording adherence. Our results can be used by designers of adherence measures to ensure future measures meet the needs of users.

The results of the survey indicate that clinicians, researchers, and older adults would like to collect data about the intensity and quality of resistance and balance exercises completed. Indeed, in our study the number of repetitions with good form and/or at a selected intensity and difficulty of balance exercises were ranked in the top three most important constructs that clinicians and researchers would like to measure, while older adults would like information on what they could improve upon, how they could improve, and what they did correctly. Our results also indicate that the parameters important to clinicians and researchers for capturing a good quality set or repetition include intensity of repetitions, challenge of the exercise, and number of repetitions. Methods for objectively collecting adherence data that captures the quality of movement for resistance and balance exercises are developing. For example, inertial measurement units have been used to measure movement smoothness for people recovering from stroke [20]. For resistance training, accelerometers can be used to measure energy expenditure during resistance exercise, [21] while smartphones [22] and resistance band integrated sensors [23] have been used to measure time under tension. Methods for objectively describing balance exercise intensity have also been developed [24]. However, these require therapists and patients to manually rate their response to a balance exercise, [25] though many of the items can be quantified and tracked using technology like camera vision (e.g., sway, ankle and hip strategy, hesitation, loss of balance). Alsubaie et al. found that rate of perceived exertion while completing balance exercises correlated with trunk angular velocity as measured by an inertial measurement unit [26]. While the field of objective quantification of balance and resistance exercise intensity is beginning to grow, we urge designers to consider the needs and preferences of users. Further design considerations for tools to measure adherence identified by older adults, clinicians, and researchers in our study include easy to use, accurate tools with clear, specific instructions that are meaningful to clients and provide real-time feedback. Older adults also wanted the ability to record all data in one place.

Our survey revealed that the most frequent current method of measuring adherence to resistance and balance exercises is a calendar or through attendance at an exercise class or session. Clinicians and researchers also most frequently ranked number of days completed as the most important item to measure for adherence. This result is consistent with a previous systematic review that found the most commonly used parameter to assess adherence was self-reported frequency of exercise (e.g., number of days per week) via exercise logs [11]. While number of days is important to gather frequency of exercise participation, it does not provide information about intensity of exercise sessions which is important to determine if a participant or client is working hard enough to achieve their goals. As discussed above, future work should continue to focus on developing systems to accurately track adherence to intensity of exercise as it was identified as important to users. However, given the importance of frequency of exercise participation in our survey results, we also recommend that future adherence measurement tools include frequency in their output.

Interestingly, most older adults in our study did not measure adherence because they did not want to. Most studies examine reasons why older adults are not adherent to exercise programs themselves, identifying items such goal setting, social influences, environment, and resources [27]. However, no work has examined the process of recording adherence from the older adults’ perspective. In our survey, we did not further examine the reasons behind not wanting to record adherence, but time, resources, motivation, and health were identified as barriers for those who did not measure adherence. While researchers often want to record adherence to determine its relationship with effectiveness of an intervention, and clinicians may be interested in understanding why clients and patients are not progressing or problem solve around issues related to adherence, the direct benefit for older adults of recording adherence remains unclear. Our work suggests that older adults’ want to know what and how they can improve their exercise, which may serve as their reason to want to measure adherence. Future work could continue to explore if and why older adults want to record exercise adherence to inform design work in this area.

A limitation of our work is that our sample size for researchers was small in comparison to the other groups and therefore may not represent all perspectives. We distributed the survey in English and German, meaning we may have missed responses in other languages and limiting the generalizability of our results to countries and settings that speak these languages. A limitation of our use of the PASE to describe physical activity levels is that it has been validated with older adults with a mean age of 70–80 years old, while our study included adults over the age of 50 years. Because this study used an online survey methodology disseminated through multiple channels (e.g., social media, professional associations), we cannot describe the response rate or the entire population to which our survey was distributed. Further, like all online surveys, respondents with biases may self-select themselves into the sample, limiting the generalizability of our results. However, it is a strength that we included participants from three stakeholder groups (older adults, clinicians, and researchers) to gain fulsome views on measuring exercise adherence. This approach was suggested by previous authors given the previously observed differences in opinions [14].

In conclusion, our work provides information about current methods of measuring exercise adherence and provides suggestions that can be used by designers of future adherence measures to ensure they meet the needs of users. We found that diaries and calendars (either analog or digital) were the most common current method of collecting adherence data. Information about the intensity and quality of exercises should be presented in clear, easy to use formats that are meaningful for older adults where all data can be tracked in one place. Most older adults did not measure adherence because they did not want to, while clinicians most frequently reported not having measurement tools for adherence. Time, resources, motivation, and health were also identified as barriers to recording adherence.

### Electronic supplementary material

Below is the link to the electronic supplementary material.

Supplementary Material 1

## Data Availability

The data analyzed in this study are not publicly available due to privacy and confidentiality restrictions pertaining to person-level health information in Canada. However, the data set creation plan and underlying analytic code are available from the corresponding author on reasonable request.

## List of abbreviations

PASE: Physical Activity Scale for the Elderly
