# Supplementary material for: Older adults, clinicians, and researchers’ preferences for measuring adherence to resistance and balance exercises
Source: BMC Geriatr. 2023 Aug 30;23:530. doi: 10.1186/s12877-023-04237-x (PMC10470185; doi:10.1186/s12877-023-04237-x)
Supplement: Supplementary file 1 — Supplementary Material 1 [file 12877_2023_4237_MOESM1_ESM.docx]

RESEARCHERS – WHO CONDUCT EXERCISE AS AN INTERVENTION STUDY

1. What is your age group?
   1. 20-29
   2. 30-39
   3. 40-49
   4. 50-59
   5. 60-69
   6. 70-79
   7. 80-89
   8. 90-99+
   9. Prefer not to respond
2. What gender do you identify as?
   1. Male
   2. Female
   3. Nonbinary
   4. Prefer not to respond

STRENGTH TRAINING ADHERENCE

1. How do you currently measure adherence to strength exercises? (check all that apply)
   1. Pen and paper calendar/diary
   2. App calendar/diary
   3. Phone call from study team – weekly or monthly
   4. Other – please specify
2. What data do you currently collect to report adherence to strength exercises? (check all that apply)
   1. Number of repetitions
   2. Number of sets
   3. Number of repetitions with good form and/or at selected intensity
   4. Number of sets with good form and/or at selected intensity
   5. Total exercise time (duration)
   6. Time to complete a set (duration)
   7. Time to complete a repetition (duration)
   8. 1 repetition max
   9. Rating of perceived exertion
   10. Number of different exercises completed
   11. Raw data – e.g., Individual joint velocities, angles, accelerations
   12. Number of days completed
   13. Other – please describe
   14. Other – please describe
   15. Other – please describe
3. What parameters do you use to define a “good” *repetition* of a strength exercise? Please rank the following from most (1) to least (6) important.
   1. Joint range of motion
   2. Joint velocity
   3. Intensity of repetitions e.g., % of 1RM
   4. Time to complete a repetition
   5. Borg Rating of Perceived Exertion Scale (RPE)
   6. Other Rating of Perceived Exertion Scale (RPE)
   7. Other – please describe
4. What parameters do you use to define a “good” *set* of a strength exercise? Please rank the following from most (1) to least (5) important.
   1. Number of repetitions
   2. Number of good repetitions
   3. Time to complete a set (duration)
   4. Intensity of repetitions e.g., % of 1RM
   5. Other – please describe
5. How do you currently track change in strength over time of your study participants?
   1. Number of repetitions
   2. Number of sets
   3. Number of repetitions with good form and/or at selected intensity
   4. Number of sets with good form and/or at selected intensity
   5. Total exercise time (duration)
   6. Time to complete a set (duration)
   7. Time to complete a repetition (duration)
   8. Intensity of repetitions e.g., % of 1RM, RPE
   9. Number of different exercises completed
   10. Raw data – e.g., Individual joint velocities, angles, accelerations
   11. Number of days completed
   12. Other – please describe
   13. Other – please describe
   14. Other – please describe
6. What barriers/facilitators do you face to measuring adherence to strength exercises? (open text box)
7. What data would you like to collect to track your study participants’ strength exercise adherence? Please rank the following from most (1) to least (11) important.
   1. Number of repetitions
   2. Number of sets
   3. Number of repetitions with good form and at selected intensity
   4. Number of sets with good form and at selected intensity
   5. Total exercise time (duration)
   6. Time to complete a set (duration)
   7. Time to complete a repetition (duration)
   8. Intensity of repetitions e.g., % of 1RM
   9. Number of different exercises completed
   10. Raw data? Individual joint velocities, angles, accelerations
   11. Number of days completed
   12. Other, please describe
   13. Other, please describe
   14. Other, please describe
8. What other data would you like to collect to track your study participant’s strength exercise adherence? (Open text box)

BALANCE TRAINING ADHERENCE

Definition: Balance training involves the efficient transfer of bodyweight from one part of the body to another or challenges specific aspects of the balance systems (e.g. vestibular systems). Balance retraining activities range from the re‐education of basic functional movement patterns to a wide variety of dynamic activities that target more sophisticated aspects of balance. (Reference: Profane taxonomy)

1. How do you currently measure adherence to balance exercises? (check all that apply)
   1. Pen and paper calendar/diary
   2. App calendar/diary
   3. Phone call from study team – weekly or monthly
   4. Other – please describe
2. What data do you collect to report adherence to balance exercises? (check all that apply)
   1. Difficulty of balance exercises (e.g., challenging vs not)
   2. Total exercise duration
   3. Number of different exercises
   4. Type of balance exercise (e.g., static vs dynamic)
   5. Number of repetitions
   6. Number of sets
   7. Number of days completed
   8. Other – please describe
   9. Other – please describe
   10. Other – please describe
3. What parameters do you use to define *challenging* balance exercises? Please rank the following from most (1) to least (4) important.
   1. Number of episodes of loss of balance during session
   2. Degree of loss of balance (e.g., wobble, taking a step, reaching out)
   3. Time to complete the session (duration)
   4. Other – please describe
4. What parameters do you use to define a “good” *session* of a balance exercises? Please rank the following from most (1) to least (5) important.
   1. Number of different balance exercises completed
   2. Types of balance exercises (e.g., static, dynamic)
   3. Challenge of the balance exercises
   4. Time to complete the session (duration)
   5. Other – please describe
5. What barriers/facilitators do you face to measuring adherence to balance exercises? (open text box)
6. What information would you like to know about your study participants’ balance exercise adherence? Please rank the following from most (1) to least (7) important.
   1. Difficulty of balance exercises (e.g., challenging vs not)
   2. Total exercise duration
   3. Number of different exercises
   4. Type of balance exercise (e.g., static vs dynamic)
   5. Number of repetitions
   6. Number of sets
   7. Number of days completed
7. What other information would you like to know about your study participant’s balance exercise adherence? (Open text box)

GENERAL INFORMATION

1. What type of mobile device would you be most comfortable using to collect exercise adherence data for your research? Please rank from most comfortable (1) to least comfortable (4).
   1. Cell phone
   2. Tablet
   3. Laptop
   4. Other – please specify
2. What specifications of the software system do you want to know?
   1. Margin of error
   2. Reliability
   3. Validity
   4. Responsiveness
   5. Other – please specify

CLINICIANS AND EXERCISE PROVIDERS – WHO PRESCRIBE STRENGTH AND BALANCE EXERCISES

1. What is your age group?
   1. 20-29
   2. 30-39
   3. 40-49
   4. 50-59
   5. 60-69
   6. 70-79
   7. 80-89
   8. 90-99+
   9. Prefer not to respond
2. What gender do you identify as?
   1. Male
   2. Female
   3. Nonbinary
   4. Prefer not to respond

STRENGTH TRAINING ADHERENCE

1. How do you currently measure your patients’ adherence to strength exercises? (check all that apply)
   1. Pen and paper calendar/diary
   2. App calendar/diary
   3. Phone call from study team – weekly or monthly
   4. Other – please specify
2. What data do you currently collect to report adherence to strength exercises? (check all that apply)
   1. Number of repetitions
   2. Number of sets
   3. Number of repetitions with good form and/or at selected intensity
   4. Number of sets with good form and/or at selected intensity
   5. Total exercise time (duration)
   6. Time to complete a set (duration)
   7. Time to complete a repetition (duration)
   8. 1 repetition max
   9. Rating of perceived exertion
   10. Number of different exercises completed
   11. Raw data – e.g., Individual joint velocities, angles, accelerations
   12. Number of days completed
   13. Other – please describe
   14. Other – please describe
   15. Other – please describe
3. What parameters do you use to define a “good” *repetition* of a strength exercise? Please rank the following from most (1) to least (6) important.
   1. Joint range of motion
   2. Joint velocity
   3. Intensity of repetitions e.g., % of 1RM
   4. Time to complete a repetition
   5. Borg Rating of Perceived Exertion Scale (RPE)
   6. Other – please describe
4. What parameters do you use to define a “good” *set* of a strength exercise? Please rank the following from most (1) to least (4) important.
   1. Number of repetitions
   2. Time to complete a set (duration)
   3. Intensity of repetitions e.g., % of 1RM
   4. Other – please describe
5. How do you currently track change in strength over time of your patients? (Select all that apply)
   1. Number of repetitions
   2. Number of sets
   3. Number of repetitions with good form and/or at selected intensity
   4. Number of sets with good form and/or at selected intensity
   5. Total exercise time (duration)
   6. Time to complete a set (duration)
   7. Time to complete a repetition (duration)
   8. 1 repetition max
   9. Rating of perceived exertion
   10. Number of different exercises completed
   11. Raw data – e.g., Individual joint velocities, angles, accelerations
   12. Number of days completed
   13. Other – please describe
   14. Other – please describe
   15. Other – please describe
6. What barriers/facilitators do you face to measuring adherence to strength exercises? (open text box)
7. What data would you like to collect to track your patients’ strength/resistance exercise adherence? Please rank the following from most (1) to least (11) important.
   1. Number of repetitions
   2. Number of sets
   3. Number of repetitions with good form and at selected intensity
   4. Number of sets with good form and at selected intensity
   5. Total exercise time (duration)
   6. Time to complete a set (duration)
   7. Time to complete a repetition (duration)
   8. Intensity of repetitions e.g., % of 1RM
   9. Number of different exercises completed
   10. Raw data-- Individual joint velocities, angles, accelerations
   11. Number of days completed
   12. Other, please describe
   13. Other, please describe
   14. Other, please describe
8. What other data would you like to collect to track your patient’s strength exercise adherence? (Open text box)

BALANCE TRAINING ADHERENCE

Definition: Balance training involves the efficient transfer of bodyweight from one part of the body to another or challenges specific aspects of the balance systems (e.g. vestibular, proprioceptive, visual systems). Balance retraining activities range from the re‐education of basic functional movement patterns to a wide variety of dynamic activities that target more sophisticated aspects of balance. (Reference: Profane taxonomy)

1. How do you currently measure adherence to balance exercises? (check all that apply)
   1. Pen and paper calendar/diary
   2. App calendar/diary
   3. Phone call from study team – weekly or monthly
   4. Other – please describe
2. What data do you collect to report adherence to balance exercises? (check all that apply)
   1. Difficulty of balance exercises (e.g., challenging vs not)
   2. Total exercise duration
   3. Number of different exercises
   4. Type of balance exercise (e.g., static vs dynamic)
   5. Number of repetitions
   6. Number of sets
   7. Number of days completed
   8. Other – please describe
   9. Other – please describe
   10. Other – please describe
3. What parameters do you use to define *challenging* balance exercises? Please rank the following from most (1) to least (4) important.
   1. Number of episodes of loss of balance during session
   2. Degree of loss of balance (e.g., wobble, taking a step, reaching out)
   3. Time to complete the session (duration)
   4. Other – please describe
4. What parameters do you use to define a “good” *session* of a balance exercises? Please rank the following from most (1) to least (5) important.
   1. Number of different balance exercises completed
   2. Types of balance exercises (e.g., static, dynamic)
   3. Challenge of the balance exercises
   4. Time to complete the session (duration)
   5. Other – please describe
5. What barriers/facilitators do you face to measuring adherence to balance exercises? (open text box)
6. What information would you like to know about your patients’ balance exercise adherence? Please rank the following from most (1) to least (7) important.
   1. Difficulty of balance exercises (e.g., challenging vs not)
   2. Total exercise duration
   3. Number of different exercises
   4. Type of balance exercise (e.g., static vs dynamic)
   5. Number of repetitions
   6. Number of sets
   7. Number of days completed
7. What other information would you like to know about your patients’ balance exercise adherence? (Open text box)

GENERAL INFORMATION

1. What type of mobile device would you be most comfortable using to collect exercise adherence data for your research or clinical practice? Please rank from most comfortable (1) to least comfortable (4).
   1. Cell phone
   2. Tablet
   3. Laptop
   4. Other – please specify
2. What specifications of the software system do you want to know?
   1. Margin of error
   2. Reliability
   3. Validity
   4. Responsiveness
   5. Other – please specify

OLDER ADULTS – WHO ENGAGE IN STRENGTH AND BALANCE TRAINING

1. What is your age group?
   1. 50-59
   2. 60-69
   3. 70-79
   4. 80-89
   5. 90-99+
   6. Prefer not to respond
2. What gender do you identify as?
   1. Male
   2. Female
   3. Nonbinary
   4. Prefer not to respond

STRENGTH TRAINING ADHERENCE – go to this section if they said they do strength training according to self-report measure.

1. How do you currently record the strength exercises you are doing? (check all that apply)
   1. Pen and paper calendar/diary
   2. App or online calendar/diary
   3. Wearable device – e.g., FitBit
   4. I don’t
   5. Other – please specify
2. (if answered “d. I don’t” to question 1) Why don’t you record your strength exercises?
   1. Not enough time
   2. I don’t want to
   3. I don’t know how to
   4. I don’t have the tools to record them
   5. Other
3. (if answered “d. I don’t” to question 1) What would help you record your strength exercises? (open text box)
4. What information about your strength exercises do you record? (check all that apply) (if said “I don’t to previous question, skip this one)
   1. Number of repetitions of each exercise (e.g., 24 squats)
   2. Number of sets of each exercise (e.g., 3 sets of 8 squats)
   3. Number of repetitions with good form and at selected intensity (e.g., 24 good squats where I felt like I was working at an intensity of 6/10 on a scale from 0-10)
   4. Number of sets and/or repetitions with good form and at selected intensity (e.g., 3 sets of 8 squats where I felt like I was working at an intensity of 6/10 on a scale from 0-10)
   5. Total exercise time (duration) (e.g., I worked out for 30 minutes)
   6. Time to complete a set (duration) (e.g., it took me 1 minute to do 1 set of squats)
   7. Time to complete a repetition (duration) (e.g., it took me 5 seconds to do 1 squat)
   8. Intensity of repetitions e.g., % of 1RM (e.g., the heaviest weight I can bench press only once is 10 pounds, I bench pressed 8 pounds while working at 80% of my heaviest lift)
   9. Number of different exercises completed (e.g., I did 10 different strength exercises)
   10. Number of days completed (e.g., I did strength training on 3 days of the week)
   11. Other – please describe
   12. Other – please describe
   13. Other – please describe
5. Where do you do your strength exercises?
   1. At the gym or community centre
   2. At a physiotherapy clinic
   3. At home
   4. A and B
   5. A and C
   6. All of the above
   7. Other – please describe
6. What makes it hard/easier for you to record how much strength training you do? (open text box)

BALANCE TRAINING ADHERENCE – go to this section if they say they do balance training according to self-report measure.

Definition: Balance training is when you move your bodyweight from one part of the body to another and challenges how well you can stay upright. During balance exercises you might feel wobbly and like you might lose your balance. Balance exercises can involve standing still (e.g., standing on one foot) or moving about (e.g., stepping over obstacles).

1. How do you currently record the balance exercises you are doing? (check all that apply)
   1. Pen and paper calendar/diary
   2. App or online calendar/diary
   3. Wearable device – e.g., FitBit
   4. I don’t
   5. Other – please specify
2. (if answered no to question 7) Why don’t you record your balance exercises?
   1. Not enough time
   2. I don’t want to
   3. I don’t know how to
   4. I don’t have the tools to record them
   5. Other
3. (if answered no to question 7) What would help you record your strength exercises? (open text box)
4. What information about your balance exercises do you record? (check all that apply)
   1. Difficulty of balance exercises (e.g., the exercise made me take a step or I had to use the wall or countertop when I lost my balance)
   2. Total exercise duration (e.g., I did 10 minutes of balance exercises)
   3. Number of different exercises (e.g., I did 8 different balance exercises)
   4. Type of balance exercise (e.g., I did balance exercises standing still (static) and moving around (dynamic)
   5. Number of repetitions (e.g., I stood on one foot for 10 seconds 5 times)
   6. Number of repetitions and sets (e.g., I stood on one foot for 10 seconds 5 times, and repeated that 3 times)
   7. Number of days completed (I did balance exercises everyday of the week)
   8. Other – please describe
5. Where do you do your balance exercises?
   1. At the gym or community centre
   2. At home
   3. Both
   4. Physiotherapy clinic
   5. Other – please describe
6. What makes it harder/easier for you to record how much balance training you do? (open text box)

GENERAL QUESTIONS

1. What type of mobile device would you be most comfortable using to track how your strength and balance exercises are going?
   1. Cell phone
   2. Tablet
   3. Laptop
   4. Other – please specify
2. When would you like feedback about how you did the exercises?
   1. During the exercise session
   2. After the exercise session
   3. Other - please specify
3. What type of feedback would you like?
   1. Verbal – the app tells you what could be improved
   2. Visual – the app shows you what could be improved
   3. Both verbal and visual feedback
   4. Other – please specify
4. What would you like feedback about? Rank from most important (1) to least important (11).
5. How many different exercises you did
6. How many repetitions you did
7. How many sets you did
8. How many days you exercised
9. Type of balance exercises you did
10. How long you exercised for
11. How many good repetitions you did
12. What you did right
13. What you could improve
14. How you can improve
15. How hard you were working
16. Other – please specify

If they do not report doing strength or balance training according to self-report measure:

1. What stops you from doing strength training? (Open text box)
2. What other types of activities do you do to keep your muscles strong? (Open text box)
3. Would you be more likely to do strength training if there were other ways to record your exercises?
   1. Yes
   2. No
   3. I don’t know
   4. Other
4. If yes to previous question, what other ways would you like to record your exercises? (open text box)
5. What stops you from doing balance training? (Open text box)
6. What other types of activities do you do to train your balance? (Open text box)
7. Would you be more likely to do strength training if there were other ways to record your exercises?
   1. Yes
   2. No
   3. I don’t know
   4. Other
8. If yes to previous question, what other ways would you like to record your exercises?
